# Anti-inflammatory effect of tranexamic acid on adult cardiac surgical patients: A PRISMA-compliant systematic review and meta-analysis

**DOI:** 10.3389/fsurg.2022.951835

**Published:** 2022-09-28

**Authors:** Chun-Mei Xie, Yun-Tai Yao, Li-Xian He, Ke Yang, the Evidence In Cardiovascular Anesthesia (EICA) Group

**Affiliations:** ^1^ Department of Anesthesiology, Fuwai Yunnan Cardiovascular Hospital, Affiliated Cardiovascular Hospital of Kunming Medical University, Kunming, China; ^2^ Department of Anesthesiology, Fuwai Hospital, National Center for Cardiovascular Diseases, Peking Union Medical College and Chinese Academy of Medical Sciences, Beijing, China

**Keywords:** cardiac surgery, tranexamic acid, inflammatory, meta-analysis, randomized controlled trials

## Abstract

**Objective:**

This study aims to evaluate the anti-inflammatory effect of tranexamic acid (TXA) on adult cardiac surgical patients.

**Methods:**

PubMed, Embase, Ovid, Web of Science, CNKI, VIP, and WANFANG databases were systematically searched using the related keywords for cardiac surgical randomized controlled trials (RCTs) published from their inception to February 1, 2022. The primary outcomes were postoperative inflammatory biomarkers levels. The secondary outcomes were postoperative systemic inflammatory response syndrome and other major postoperative outcomes. The odds ratios and/or the weighted mean difference (WMD) with a 95% confidence interval (CI) were used to pool the data.

**Results:**

Ten RCTs with 770 adult cardiac surgical patients were included. Compared with placebo, TXA achieved statistically significant inhibition of the postoperative interleukin (IL)-6 level (postoperative 6 h: *n* = 6 trials; WMD −31.66; 95% CI: −45.90, −17.42; *p *< 0.0001; *I*^2 ^= 93%; postoperative 24 h: *n *=  8 trials; WMD, −44.06; 95% CI: −69.21, −18.91; *p *= 0.006; *I*^2 ^= 100%); IL-8 level postoperative 24 h, TNF-α level postoperative 24 h, NE level postoperative 6 h: *n* = 3 trials; WMD, −36.83; 95% CI: −68.84, −4.83; *p *= 0.02; *I*^2 ^= 95%); tissue necrosis factor alpha (TNF-α) level (postoperative 6 h: *n* = 3 trials; WMD, −7.21; 95% CI: −12.41, −2.01; *p *= 0.007; *I*^2 ^= 47%; postoperative 24 h: *n* = 5 trials; WMD, −10.02; 95% CI: −14.93, −5.12; *p *< 0.0001; *I*^2 ^= 94%); and neutrophil elastase (NE) level (postoperative 6 h: *n* = 3 trials; WMD, −66.93; 95% CI: −111.94, −21.92; *p *= 0.004; *I*^2 ^= 86%). However, TXA achieved no statistically significant influence on the postoperative 24 h NE level.

**Conclusions:**

TXA had a significant anti-inflammatory effect in adult cardiac surgical patients, as evidenced by the reduction of multiple postoperative proinflammatory biomarkers levels, but these results should be interpreted carefully and cautiously, as only a limited number of studies were included and there was high heterogeneity between them.

**Systematic Review Registration:**

https://www.crd.york.ac.uk/prospero/#recordDetails, identifier: CRD42022312919.

## Introduction

Surgery is known to cause tissue damage, and initiate inflammatory response (1), particularly cardiac surgery with cardiopulmonary bypass (CPB) (2, 3). The inflammatory response characterized by the release of proinflammatory cytokines (4, 5) may cause a hypotension/hypoperfusion state (6). Elevations in IL-6 and IL-8 levels after CPB were associated with an increased risk of organ injury (7–10) and mortality (9, 11). Numerous strategies to reduce inflammatory response and bleeding in cardiac surgical patients exist, among which is the use of tranexamic acid (TXA). TXA is a traditional antifibrinolytic drug, fibrinolysis is a marker for the onset of systemic inflammation (12), and plasmin inhibition can mitigate immunosuppression after certain ischemic events including surgery (13). Simultaneously, Cvachovec et al.'s study (14) summarized the multifaceted role of fibrinogen in tissue injury and inflammation and found that the universal presence of fibrin within inflammatory foci, similarly to the extravascular fibrin deposits, exacerbates inflammation across a spectrum of disease models. Casati et al. reported that TXA significantly reduced bleeding in coronary artery bypass grafting (CABG) and may modulate inflammation in these surgical settings (15). In addition, another study showed that TXA exhibited a minor anti-inflammatory response (16). Inversely, Later et al. reported that aprotinin attenuated the postoperative TNF-α level, whereas TXA did not, and the majority of plasma cytokines (IL-6, IL-8, and IL-10) were not affected by the use of antifibrinolytics when compared with placebo (17). In addition, TXA treatment attenuated the surgery-induced increase in the level of proinflammatory cytokine IL-1β, but it did not significantly alter the levels of TNF-α, IL-6, IL-8, and IL-10 (18).

Therefore, the inflammatory effect of TXA in adult cardiac surgical patients remains controversial. We conducted a systematic review and meta-analysis of randomized controlled trials (RCTs) with the aim of evaluating the anti-inflammatory effect of TXA in adult cardiac surgical patients.

## Methods

This study followed the methodology outlined in the Cochrane Handbook for Systematic Reviews of Interventions Version 6.0 (19). We explained it in accordance with the Preferred Reporting Items for Systematic Reviews and Meta-Analyses Protocols statement. This protocol has been registered on the International Prospective Systematic Reviews Registry database (PROSPERO: CRD42022312919).

### Systematic search

We conducted a comprehensive search of PubMed, Embase, Web of Science, CNKI, WANFANG, VIP, and unpublished sources including ClinicalTrials.gov, ChiCTR, and the Cochrane trial registry from inception to February 1, 2022, for RCTs investigating the role of TXA in adult patients undergoing cardiac surgery. Language was limited to English and Chinese; the related searching words were as follows: (tranexamic acid) OR (TXA) AND [(inflammatory) OR (cytokine)] AND [(cardiac surgery) OR (cardiopulmonary bypass) OR (coronary artery bypass surgery) OR (valve surgery) OR (aortic surgery) OR (congenital heart disease)] AND (randomized controlled trial OR controlled clinical trial OR randomly OR trial) in the title/abstract. In addition, we manually searched the references of the identified studies to identify further relevant studies.

### Study selection

The study selection criteria are as follows: (1) Population: Population of interest were adult patients undergoing cardiac surgery. Studies concerning children, infants, or newborns were excluded. (2) Intervention: The intervention group was TXA administration. (3) Comparator: The intervention group was compared with the placebo group. (4) Outcome: The postoperative inflammatory biomarkers levels were included. The inflammatory biomarkers chosen as outcomes were cytokines IL-6, IL-8, TNF-α, and NE. Time points for cytokine measurement were grouped into 6 h postoperatively and 24 h postoperatively. We chose these biomarkers and time points to align with the outcomes most commonly used in identified relevant studies. To limit heterogeneity across sampling periods, we excluded studies that measured inflammatory biomarkers outside our specified time points. (5) Study design: We only included RCTs to ensure that the combined results were of good quality and excluded the studies that could not provide effective analysis data.

After implementing the search strategy, two researchers (C-MX and Y-TY) screened all potentially relevant citations independently and in duplicate. Citations deemed potentially relevant by either screener were advanced to second-stage full-text review. Full texts were subsequently reviewed for eligibility, with disagreements resolved by consensus and third-party adjudication if required. Trials were excluded for not reporting the results of the marker of interest.

### Data extraction and quality assessment

Reviewers (C-MX, L-XH, and KY) extracted data independently and in duplicate using prepiloted data abstraction forms. The extracted data are as follows: the first author, published year, demographic data, details of the intervention and placebo, surgical procedure, inflammatory biomarkers levels, modified *Jadad* score, and risk of bias for each study. Reviewers (C-MX, L-XH, and KY) examined the following risk of bias domains: randomized sequence generation, allocation concealment, blinding, incomplete outcome, selective reporting, and other bias (such as stopping early and funding sources).

### Statistical analysis

All data were analyzed by Review Manager 5.4 (Cochrane Collaboration, Oxford, UK). The odds ratios with 95% confidence intervals (CIs) were estimated for dichotomous data, and weighted mean differences (WMDs) with 95% CIs were estimated for continuous data. If fewer than three studies reported a specific outcome and time point, these data were not pooled. Each outcome was tested for heterogeneity, and the randomized-effects model or fixed-effects model was used in the presence or absence of significant heterogeneity, Q-statistical test *p *< 0.05, and *I*^2^ statistics (*I*^2^ > 50% was considered as the presence of significant heterogeneity). Sensitivity analyses were performed by examining the influence of the statistical model on estimated treatment effects, and analyses that adopted the fixed-effects model were repeated again by using the randomized-effects model and vice versa. In addition, sensitivity analysis was also performed to evaluate the influence of individual studies on the overall effects. Subgroup analyses were performed to evaluate the possible effects of patient characteristics and control agents on the outcomes, if necessary. Publication bias was explored through visual inspection of funnel plots of the outcomes. All *p* values were two-sided, and statistical significance was defined as *p *< 0.05.

## Results

### Literature search results

**Figure 1:**
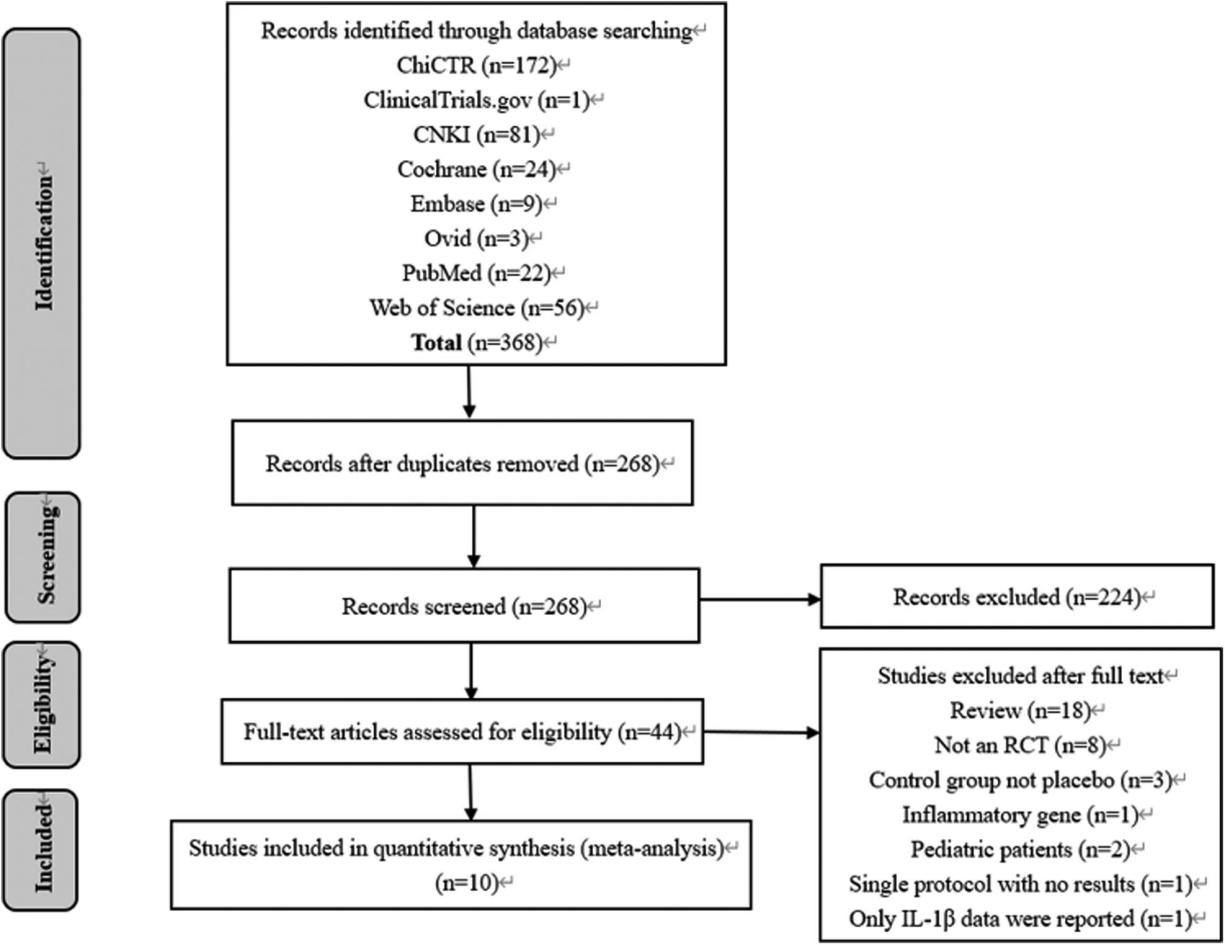
Study flowchart.

As depicted in the flowchart (Figure 1), our initial search yielded 368 records. A total of 324 trials were excluded by being duplicated and reviewing the titles and abstracts. In total, 44 full texts were assessed, and finally, 10 RCTs with 770 adult cardiac surgical patients were included in this meta-analysis (15–17, 20–26).

### Characteristics of included studies

Baseline characteristics of included trials are summarized in Table 1. One RCT was registered in the study (20), and seven studies were conducted in China (16, 21–26). The dosage of TXA was imparity across included trials; similarly, the timing and method of TXA administration varied among studies: six studies selected loading dose and continuous infusion (15–17, 23, 25, 26), while other studies chose a time point for the injection of TXA (20–22, 24).

**Table 1:** Characteristics of the included trials.

Study	Country	*N*_Total_	Group	*N*_TXA_	*N*_Placebo_	TXA dose	Biomarkers assessed	Surgical procedure
Casati et al. (15)	Italy	102	2	26	25	Bolus: 1,000 mg + maintenance: 400 mg/h	IL-6	Off-pump CABG
				26	25	Bolus: 1,000 mg + CPB: 500 mg + maintenance: 400 mg/h	IL-6	On-pump CABG
Chen et al. (16)	China	60	1	30	30	Bolus: 15 mg/kg + maintenance: 15 mg/kg/h	IL-6, TNF-α, NE	VR
Jimenez et al. (20)	Spain	50	1	24	26	Bolus: 2,000 mg	IL-6	Elective CPB surgery
Later et al. (17)	Netherlands	17	1	8	9	Bolus: 1,500 mg + CPB: 500 mg + maintenance: 400 mg/h	IL-1α, IL-1β, IL-2, IL-4,	On-pump
							IL-6, IL-8, IL-10, IFN-α	CABG/VR/combination
Li et al. (21)	China	40	1	20	20	Bolus: 10 mg/kg at induction + CPB end + Surgery end	IL-6	VR/ASDR/VSDR
Chuan-bin (22)	China	60	1	30	30	Maintenance: 80 mg/kg during CPB	IL-6, TNF-α	VR
Lv et al. (23)	China	101	1	20	20	Bolus: 10 mg/kg + maintenance: 10 mg/kg/h	NE	On-pump CABG/VR/combination
Shi (24)	China	200	1	100	100	Bolus: 15 mg/kg after heparin + after protamine	IL-6, IL-8, IL-10, TNF-α, NE	CHD/On-pump CABG/Valve
Wang et al. (25)	China	60	1	30	30	Bolus: 1,000 mg + 400 mg/h iv infusion	IL-6	Off-pump CABG
Yu (26)	China	80	3	20	6	Bolus: 30 mg/kg + maintenance: 20 mg/kg/h	TNF-α	VR
				20	7	Bolus: 20 mg/kg + maintenance: 15 mg/kg/h	TNF-α	VR
				20	7	Bolus: 10 mg/kg + maintenance: 10 mg/kg/h	TNF-α	VR

Bypass. IFN-α, interferon alpha; IL, interleukin; NE, neutrophil elastase; TNF-α, tissue necrosis factor-alpha; VR, valve repair or replacement; VSDR, ventricular septal defect repair. ASDR, atrial septal defect repair; CABG, coronary artery bypass grafting; CHD, congenital heart disease; CPB, cardiopulmonary.

### Risk of bias in included studies

**Figure 2:**
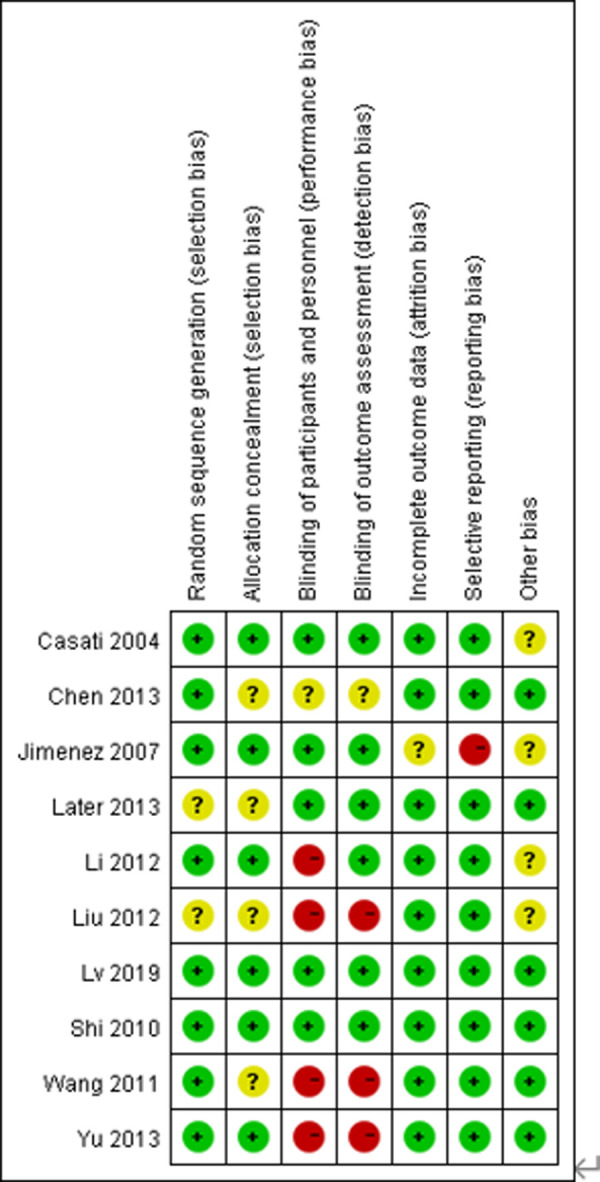
Risk-of-bias graph for each included study. Green (+), red (–), and yellow (?) circles indicate low, high, and unclear risk of bias, respectively.

**Figure 3:**
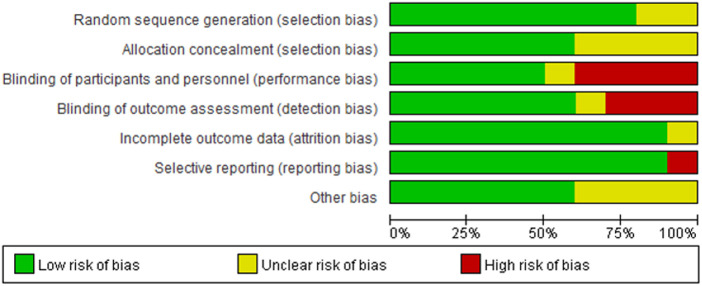
Risk-of-bias summary for each included study. Green (+), red (–), and yellow (?) circles indicate low, high, and unclear risk of bias, respectively.

Details regarding the performance of the studies against each domain were presented in the risk of the bias graph (Figure 2). In addition, a visual summary of judgments about each methodological quality item for each included trial is given in Figure 3. Of the 10 included trials, the modified *Jadad* score for the 3 studies was three points (21, 22, 26), and these studies were considered lowquality studies, as shown in Table 2.

**Table 2:** Quality assessment of included studies.

Study	Sample size	Modified Jadad score				
		Randomization	Allocation	Blindness	Withdrawals	Total
Casati et al. (15)	102	2	2	2	0	6
Chen et al. (16)	60	2	1	2	0	5
Jimenez et al. (20)	50	2	2	2	0	4
Later et al. (17)	17	1	1	2	0	3
Li et al. (21)	40	1	0	2	0	3
Chuan-bin et al. (22)	60	1	0	2	0	3
Lv et al. (23)	101	2	2	2	0	6
Shi (24)	200	1	1	2	0	4
Wang et al. (25)	60	2	1	2	0	5
Yu (26)	80	1	0	2	0	3

### Primary outcomes

**Figure 4:**
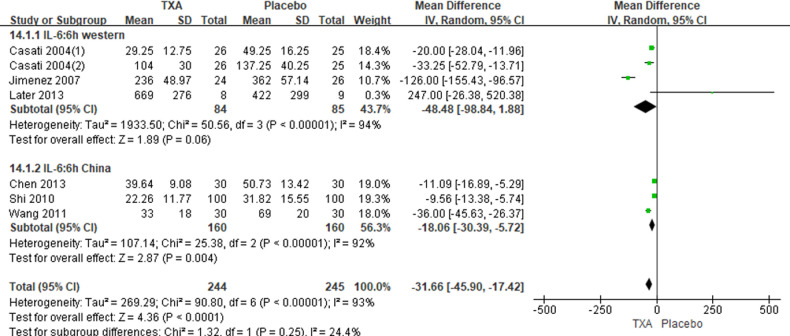
Forest plot comparing TXA and placebo for the postaperative 6 h IL-6 level.

**Figure 5:**
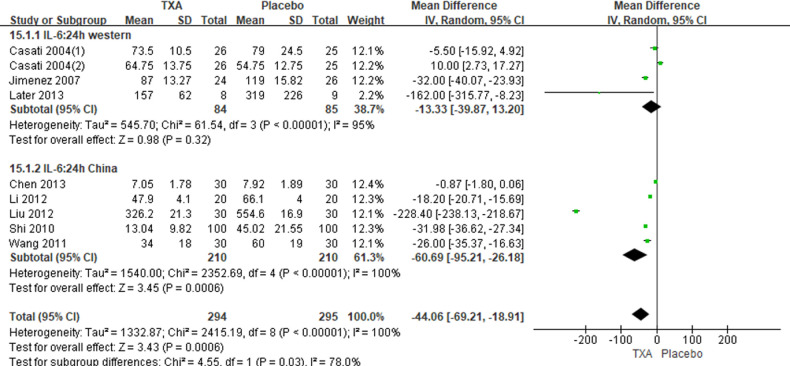
Forest plot comparing TXA and placebo for the postaperative 24 h IL-6 level.

**Figure 6:**
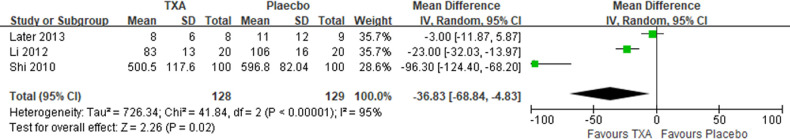
Forest plot comparing TXA and placebo for the postaperative 24 h IL-8 level.

**Figure 7:**
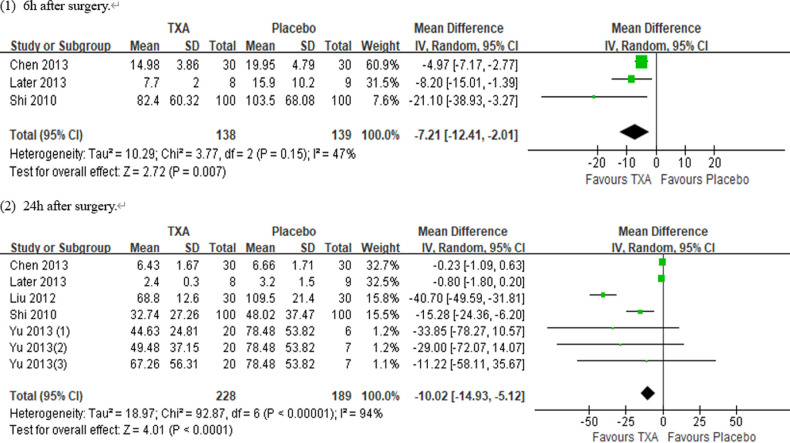
Forest plot comparing TXA and placebo for the TNF-α level.

**Figure 8:**
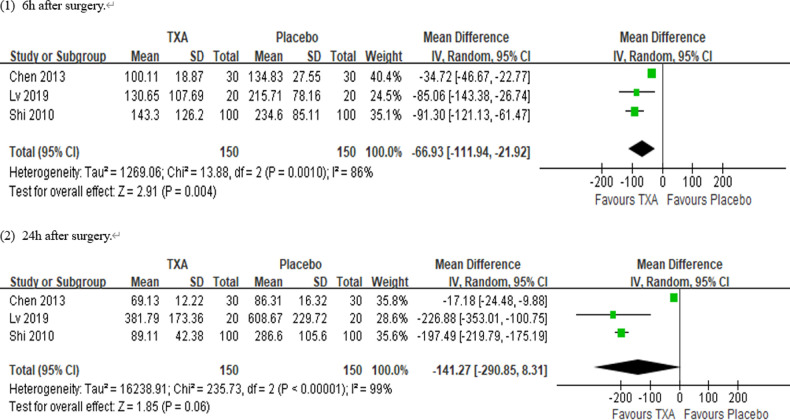
Forest plot comparing TXA and placebo for the neutrophil elastase (NE) level.

In total, 10 RCTs with 770 cardiac surgical adult patients were included. Compared with placebo, TXA achieved statistically significant inhibition of the postoperative IL-6 level (6 h: *n* = 6 trials; WMD, −31.66; 95% CI: −45.90, −17.42; *p *< 0.0001; *I*^2 ^= 93%; 24 h: *n* = 8 trials; WMD, −44.06; 95% CI: −69.21, −18.91; *p *= 0.006; *I*^2 ^= 100%) (Figures 4, 5), IL-8 level postoperative 24 h, TNF-α level postoperative 24 h, NE level postoperative 6 h: *n* = 3 trials; WMD, −36.83; 95% CI: −68.84, −4.83; *p *= 0.02; *I*^2 ^= 95%) (Figure 6), TNF-α level (6 h: *n* = 3 trials; WMD, −7.21; 95% CI: −12.41, −2.01; *p *= 0.007; *I*^2 ^= 47%; 24 h: *n* = 5 trials; WMD, −10.02; 95% CI: −14.93, −5.12; *p *< 0.0001; *I*^2 ^= 94%) (Figure 7), and NE level (6 h: *n* = 3 trials; WMD, −66.93; 95% CI: −111.94, −21.92; *p *= 0.004; *I*^2 ^= 86%) (Figure 8). However, TXA achieved no statistically significant influence on the postoperative 24 h NE level (Figure 8).

Only two studies reported the postoperative IL-1β level (17, 18), IL-10 level (17, 24), and postoperative 6 h IL-8 level (17, 24); therefore, these time point biomarkers were not included in this study.

In the figures, RCTs were listed in order by the name of the author. The size of each square denoted the weight of each trial's WMD in calculating the summary estimate for the overall effect on IL-6, IL-8, TNF-α, and NE. The diamond represented the summary estimate for the combined WMD at the center; opposing points of the diamond represented the 95% CIs. Three diamonds in each section represented high, low, and overall effects.

### Secondary outcomes

Jimenez et al. (20) reported that inflammatory response was found in 26 (33%) of 79 patients who did not receive TXA vs. 8 (9%) of 86 patients who received TXA, and another study (17) shown that systemic inflammatory response syndrome (SIRS) was found in all patients in the placebo group (*n* = 9) and TXA group (*n* = 8). Jimenez et al. (20) reported that 20 (12%) of the 165 patients presented vasoplegic shock. In the non-TXA group, 16 (20%) out of 79 patients developed vasoplegic shock. As expected, patients with inflammatory response were more likely to develop vasoplegic shock (58% vs. 0%; *p *< 0.001).

### Sensitivity analysis and publication bias

**Figure 9:**
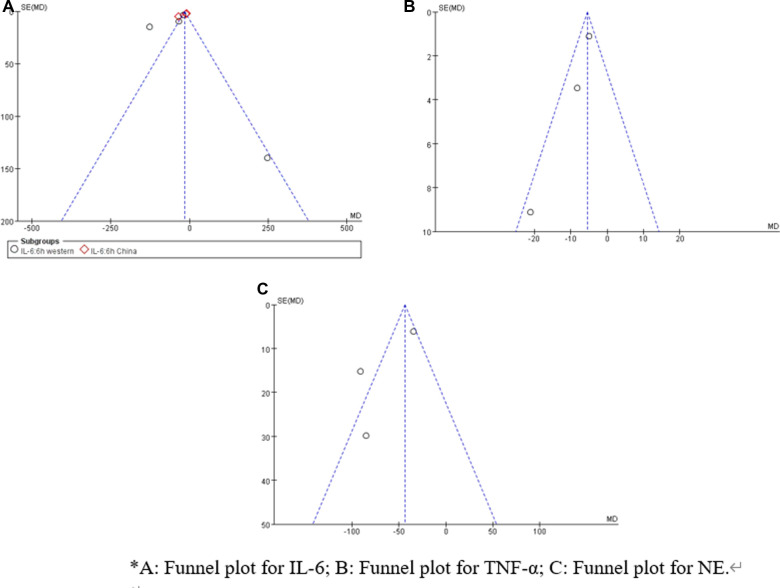
Funnel plot examination for postoperative 6 h inflammatory biomarkers.

**Figure 10:**
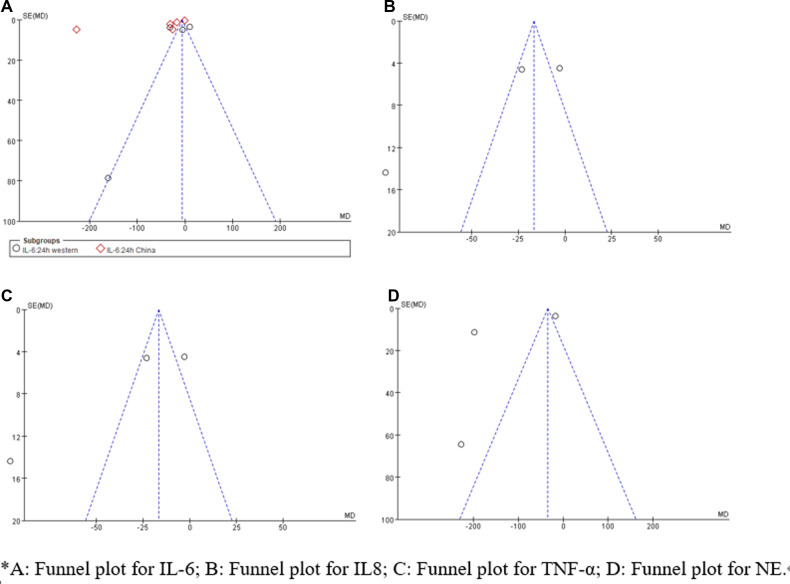
Funnel plot examination for postoperative 24 h inflammatory biomarkers.

Sensitivity analysis showed that treatment effects on all the outcomes were not affected by choice of the statistical model (Tables 3, 4). Sensitivity tests were also performed by the exclusion of some studies to analyze the influence of the overall treatment effect on high-heterogeneity outcomes (Table 5), but no contradictory results were found. Otherwhile, in Figures 9, 10, we found that there may be little publication bias.

**Table 3:** Influence of statistical model on TXA efficacy of inflammatory biomarker IL-6.

Subgroup	Statistical model	Post-op 6h: IL-6, pg/ml. WMD (95% CI)	Post-op 24h: IL-6, pg/ml. WMD (95% CI)
Studies from other countries	Random effects	−18.06 (−30.39, −5.72)	−13.33 (−39.87, 13.20)
	Fixed effects	−12.59 (−15.62, −9.57)	−60.69 (−95.21, −26.18)
Studies from China	Random effects	−31.66 (−45.90, −17.42)	−8.27 (−13.06, −3.47)
	Fixed effects	−27.97 (−35.17, −20.76)	−5.84 (−6.69, −4.99)

^a^ 95% CI, 95% confidence interval; IL-6, interleukin-6; Post-op, postoperative; WMD, weighted mean difference.

**Table 4:** Influence of statistical model on TXA efficacy of inflammatory biomarkers IL-8, TNF-α, and NE.

Statistical model	Post-op 6 h: TNF-α, NE, pg/ml. WMD (95% CI)		Post-op 24 h: IL-8, TNF-α, NE, pg/ml. WMD (95% CI)		
	TNF-α	NE	IL-8	TNF-α	NE
Random effects	−7.21 (−12.41, −2.01	−66.93 (−111.94, −21.92)	−36.83 (−68.84, −4.83)	−10.02 (−14.93, −5.12)	−141.27 (−290.85, 8.31)
Fixed effects	−5.49 (−7.57, −3.41)	−44.03 (−54.92, −33.13)	−16.85 (−23.03, −10.68)	−0.77 (−1.42, −0.13)	−35.19 (−42.11, −28.27)

Post-op, postoperative; WMD, weighted mean difference. ^a^95% CI, 95% confidence interval; IL-8, interleukin-8; TNF-α, tumor necrosis factor alpha; NE, neutrophil elastase.

**Table 5:** Sensitivity analyses of high-heterogeneity outcomes.

Heterogeneity outcome	Excluded trials	Group TXA (*n*)	Group placebo (*n*)	Heterogeneity		Analysis model	WMD	95% CI	Overall effect *P*
				*I* ^2^	*P*				
Post-op 6 h IL-6	(20, 25)	190	189	70%	<0.0001	IV, Fixed	−11.84	(−14.78, −8.91)	0.005
Post-op 24 h IL-6	(22)	240	239	98%	<0.00001	IV, Fixed	−3.95	(−4.80, −3.11)	<0.00001
Post-op 24 h IL-8	(24)	28	29	90%	0.002	IV, Fixed	−12.82	(−19.15, −6.49)	<0.0001
Post-op 24 h TNF-α	(22)	198	159	66%	0.01	IV, Fixed	−0.56	(−1.21, 0.09)	0.09
Post-op 6 h NE	(16)	120	120	0%	0.85	IV, Fixed	−90.01	(−116.57, −63.45)	<0.00001
Post-op 24 h NE	(16)	120	120	0%	0.65	IV, Fixed	−198.38	(−220.34, −176.42)	<0.00001

Post-op, postoperative; WMD, weighted mean difference. 95% CI, 95% confidence interval; IL, interleukin; TNF-α, tumor necrosis factor-alpha; NE, neutrophil elastase.

## Discussion

Surgery-associated tissue damage stimulates systemic inflammatory cascades to induce a surge in the release of cytokines and stress hormones and leukocyte migration to the injury site. The excessive inflammatory responses not only leave deleterious effects on wound healing but also is thought to cause a series of complications, such as postoperative pain, fatigue, atrial fibrillation, acute kidney injury, and cognitive dysfunction (27–29). In the present meta-analysis, we found a significant decrease in the concentrations of IL-6, IL-8, TNF-a, and NE after TXA administration in adult cardiac surgical patients, which is indicative of the anti-inflammatory potentials of TXA. Together, these data provide evidence that TXA exerts an anti-inflammatory effect and attenuates perioperative inflammation of adult cardiac surgical patients.

Some underlying mechanisms have been discussed. First, TXA is a traditional antifibrinolytic drug. Fibrinolysis is a marker for the onset of systemic inflammation (12), and plasmin inhibition can mitigate immunosuppression after certain ischemic events such as surgery (13). Second, cytokines themselves can cause some typical clinical symptoms such as fever, which involves IL-1, IL-6, TNF-α, IL-1Ra, and IL-10. Third, blood transfusion in surgery has been identified as an independent predictor of increased infection (30). Inflammation influenced the initiation and propagation of blood coagulation (31). TXA had reduced perioperative blood loss and transfusion requirements in cardiac surgical patients (32–37).

Coagulation, fibrinolysis, and inflammation are closely interconnected. As seen in Table 6, 7 of the 10 RCTs included in this study demonstrated the association between the anti-inflammatory effects of TXA and patients' clinical outcomes (e.g., bleeding, transfusion, and postoperative recovery).

**Table 6:** Inflammation and clinical outcomes of TXA administration.

Study	Inflammation					Outcomes					Mechanisms
	NE	IL-6	IL-8	IL-10	TNF-α	Bld	Tx	MVD	LOSICU	LOSH	
Casati et al. (15)	** **	**↓**	** **	** **	** **	**↓**	**↓**	(**−**)	(**−**)	(**−**)	TXA protects platelet function. Positive feedback between inflammation and coagulation, control of inflammation may reduce postoperative hypercoagulability
Chen et al. (16)	**↓**	**↓**	** **	** **	**↓**	**↓**	** **	(**−**)	(**−**)	** **	Inflammation is closely related to hemostatic alterations, attenuate inflammatory changes through blockade of fibrinolysis
Jimenez et al. (20)					**↓**	**↓**	**↓**	**↓**	(**−**)	(**−**)	Inflammation and bleeding could be considered as final outcomes of the same triggering stimulus, so that hyperfibrinolysis could play an important role in these processes
Later et al. (17)		**↓**	**↓**	**↓**	**↓**						Not mentioned
Li et al. (21)		**↓**	**↓**								Not mentioned
Chuan-bin (22)		**↓**	**↓**						**↓**	**↓**	Not mentioned
Lv et al. (23)	**↓**					**↓**	**↓**				TXA inhibits plasmin activation and protects platelet function. The inhibitory effect of TXA on the release of proinflammatory cytokines may be related to the reduction of plasminogen activation and the inhibition of hyperfibrinolysis
Shi (24)	**↓**	**↓**	**↓**	**↑**	**↓**	**↓**	**↓**		(**−**)	—	Activation of the coagulation system is an important component of the acute inflammation
Wang et al. (25)		**↓**				**↓**	**↓**				TXA inhibits fibrinolytic activation, reduces postoperative bleeding and inflammation
Yu (26)					**↓**	**↓**	**↓**		(**−**)	(**−**)	TXA inhibits plasminogen activation and hyperfibrinolysis. TXA protects platelet function

Bld, bleeding; LOSH, length of stay in hospital; LOSICU, length of stay in the intensive care unit; MVD, mechanical ventilation duration; Tx, transfusion; (−), make no difference; ↓, reduce; ↑, increase.

Among this study, IL-6 had been reported in eight studies (15–17, 20–22, 24, 25), so the postoperative IL-6 level was probably the most trustworthy. IL-6 is one of the main proinflammatory cytokines (38) and is widely recognized to play an important role in mediating the systemic inflammatory response to cardiac surgery with CPB (39). The patients aged >70 years undergoing cardiac operations generate more IL-6 during CPB (40). A multicenter cohort study of adults undergoing CABG demonstrated that an elevated level of postoperative IL-6 was associated with a higher risk of readmission and mortality (41). Other studies reported that IL-6 had a good predictive value for 30 days and overall mortality in the cardiac surgery population (42, 43). Meanwhile, numerous studies have demonstrated that elevated IL-6 level was associated with cardiac events, including incidence of heart failure, unstable angina, acute kidney injury, and functional status outcomes for patients after cardiac surgery (44–47). In this study, TXA significantly reduced the expression of postoperative IL-6, which may decrease the incidence of complications associated with an elevated level of postoperative IL-6.

As we all know, meta-analysis could increase the power of analysis by pooling many small, low-quality studies, but there was heterogeneity in the included studies. While these studies were comparable based on their design and outcomes, heterogeneity affected the validity of pooled results. In addition, high heterogeneity has been reported in previous systematic reviews. It indicated that the impact of patient and surgical variables, both measured and unmeasured, on the biomarker response likely far outweighs the impact of agents (48). Race subgroup analysis was performed in this study; however, no exact reason for the observed heterogeneity was determined.

## Limitations

The limitations of this study should be acknowledged. First, SIRS is a complex pathophysiology process influenced by multiple factors (49); however, this study only analyzes the concentrations of proinflammatory and anti-inflammatory cytokines to reflect SIRS severity. Second, the sample size of this study might be insufficient (the sample size of most articles is less than 100). Third, included patients of this study are not homogeneous. For example, 7 studies enrolling 601 patients were conducted in China; 3 studies enrolling 169 patients were conducted in western countries; 4 studies enrolling 240 patients undergoing valve replacement/repair or congenital heart disease repair; and 2 studies enrolling 162 patients undergoing CABG, particularly the OPCABG patients could exclude SIR caused by CPB and the return of shed blood into the circulation. Fourth, the most recent RCTs included in our meta-analysis were conducted in 2013, and many perfusion techniques have been adapted since then. For example, average hematocrit during CPB rose from 23% in 2005 to 30% nowadays (hemodilution has been associated with inflammation and bleeding). Fifth, the dosage, time point, and administration of TXA were diverse, and these data were not suitable for subgroup analysis, which may be the source of heterogeneity. Finally, publication bias may exist.

## Conclusions

TXA had a significant anti-inflammatory effect in adult cardiac surgical patients, as evidenced by the reduction of multiple postoperative proinflammatory biomarker levels, but these results should be interpreted carefully and cautiously, as only a limited number of studies were included and there was high heterogeneity between them.

## Data Availability

The original contributions presented in the study are included in the article/Supplementary Material, further inquiries can be directed to the corresponding author.
